# Insurance directed conservative care for low back pain: A prospective observational study

**DOI:** 10.1016/j.inpm.2026.100741

**Published:** 2026-02-13

**Authors:** David Sherwood, Margaret Helen Rutherford Riser, Peter Chia Yeh, Augustine C. Lee, Byron Schneider

**Affiliations:** a Department of Orthopedics, University Health, Lakewood Medical Center, Kansas City, MO, USA; b Vanderbilt University Medical School, Nashville, TN, USA; c OrthoVirginia, Alexandria, VA, USA; d Post-Acute Physicians of Arizona, Peoria, AZ, USA; e Department of Physical Medicine & Rehabilitation, Vanderbilt University Medical Center, Nashville, TN, USA

**Keywords:** Low back pain, Insurance, Conservative management, Physical therapy, Medication

## Abstract

**Background:**

Low back pain (LBP) is commonly managed under insurance-directed care models that mandate a trial of conservative treatment before authorizing advanced imaging or interventional procedures. This study prospectively evaluates the clinical outcomes of a six-week care program as mandated by insurers.

**Methods:**

New adult patients with LBP presenting to an academic spine clinic were enrolled. Exclusion criteria included cervical complaints, worker's compensation, and litigation. Patients received treatment pathways based on physician discretion and patient adherence. Patients were categorized into four treatment pathways at their 6-week follow up: Medication + Therapy, Medication Only, Therapy Only, or Neither. Primary outcomes were Numeric Rating Scale (NRS) for pain and Oswestry Disability Index (ODI) at 6 weeks.

**Results:**

Ninety-nine patients (mean age 57.8 years; duration of pain 52 months) completed the study. At 6 weeks, pathways including medication (Medication + Therapy and Medication Only) showed modest analgesic improvement (NRS change −1.2 and −1.8, respectively). The Therapy Only group (n = 9) showed minimal change from a lower baseline severity. Among patients prescribed physical therapy (PT), those who attended (n = 37) achieved superior 6-week scores in pain (3.8 vs 4.4, p = 0.04) and disability (20.5 vs 25.7) compared to non-attendees, though attendees had lower baseline severity. Higher PT session counts were not associated with greater symptom relief. Opioid use (n = 12) was not associated with improved outcomes.

**Conclusion:**

Mandated conservative care produced only modest improvement in pain and/or function over 6 weeks. While medication inclusion provided consistent relief, PT attendance, rather than session frequency, was the primary factor associated with better functional status. These data support setting realistic expectations for early conservative management.

## Highlights

• Insurance often mandates 6 weeks of conservative care before advanced spinal imaging.
• We followed 99 patients undergoing these mandated conservative care pathways.
• Medication-inclusive regimens yielded modest short-term pain reduction.
• Physical therapy adherence was associated with better outcomes than non-adherence and lower baseline severity.

## 1 Introduction

Private insurance companies contracted by the Center for Medicare & Medicaid Services (CMS) guide chronic low back pain (LBP) care by establishing coverage policies that define which treatments and tests are considered medically necessary^1^. These policies, which vary by geographic region, are intended to ensure a stepwise, cost-effective approach to care. Before approving advanced imaging, procedures, or operations, insurers often require a minimum of six weeks of conservative care. While the precise duration and definition of conservative care vary by insurer, it typically encompasses a structured physical therapy regimen, a provider-supervised home exercise program, and non-opioid medication. If symptoms persist despite adherence to these measures, further diagnostic workup or escalation of care is typically authorized.^2–11^ While these guidelines intend to standardize care and minimize costs, it is unclear in practice to what extent these guidelines alter symptoms. This study prospectively evaluates the clinical outcomes of patients treated under these constraints.

## 2 Methods

### 2.1 Enrollment and study design

This study was approved by the Vanderbilt University Medical Center Institutional Review Board IRB # 211219. Consecutive new patients were screened over a two-month enrollment period. Informed consent was obtained from all participants in a private setting prior to enrollment.

### 2.2 Inclusion criteria

New adult patients (≥18 years) presenting to the Vanderbilt PM&R Spine Clinic for low back pain (defined as pain below the neck) were eligible for inclusion.

### 2.3 Exclusion criteria

Patients were excluded if they [1]: were under 18 years of age [2], had prior evaluations at the Vanderbilt Spine Clinic (including Orthopedic Surgery, Neurosurgery, Interventional Pain, or another PM&R physician) [3], presented with a chief complaint involving the cervical spine or neck [4], had an active worker's compensation claim [5], were involved in active or pending litigation [6], were currently incarcerated [7], or were non–English speaking.

### 2.4 Study procedures

#### 2.4.1 Baseline assessment (Day 1)

Participants underwent a structured assessment that included [1]: patient interview and discussion with a PM&R physician [2], documentation of prior diagnostic tests and treatments in the past 12 months [3], assessment of patient expectations regarding diagnostic workup and treatment [4], duration of current pain complaint [5], pain severity measured using the Numeric Pain Rating Scale (NPRS, 0–10) [6], anticipated pain reduction over the 6 weeks [7], Global Perceived Improvement (GPI) rating (scale: 5 to +5) [8], Oswestry Disability Index (ODI) assessment [9], physician prediction of GPI at 6 weeks [10], recommended treatment documentation [11], collection of demographic data via electronic medical records.

#### 2.4.2 Follow-up assessment (Day 42–56)

Follow-up data were collected via telephone and/or email using REDCap. Collected data included [1]: treatments pursued during the preceding 6 weeks [2], GPI rating [3], Pain severity (NPRS) [4], Perceived pain reduction [5], ODI score.

## 3 Results

### 3.1 Enrollment

**Fig. 1:**
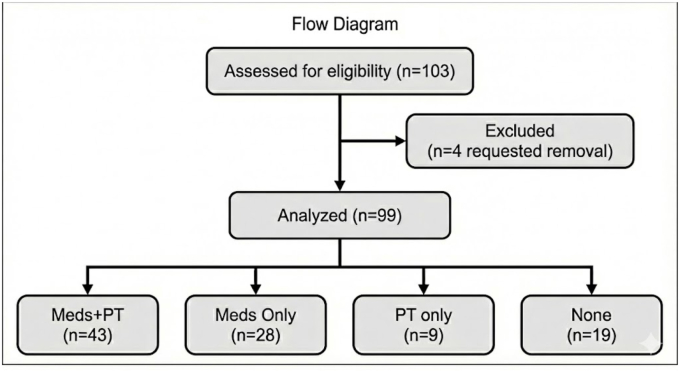
Enrollment flow diagram.

Of 103 patients enrolled, 4 requested removals from the study, yielding a final analytic sample of 99 patients. See Fig. 1.

### 3.2 Demographics

A total of 99 participants were included for this study with a mean age of 57.8 years ± 12.1, BMI 29.7 ± 6.4, and baseline duration of complaint averaged 52.0 months ±86.8. There was a nearly equal sex distribution (52.9% male, 47.1% female). Most were White (88.2%), with smaller proportions identifying as Black (5.9%), Hispanic (1.5%), or Asian (1.5%). The mean baseline pain score was 5.1 ± 2.4, and Oswestry Disability Index score was 29.7 ± 6.4 (See Table 1).

**Table 1:** Demographics and baseline characteristics.

	Overall	Therapy + Meds	Meds only	Therapy only	No treatment
N	99	43	28	9	19
Age, years (mean ± SD)	57.8 ± 16.3	60.9 ± 14.9	52.6 ± 17.7	60.9 ± 13.1	57.1 ± 17.4
BMI, kg/m^2^ (mean ± SD)	29.7 ± 6.4	29.3 ± 5.8	30.8 ± 8.0	27.2 ± 5.5	30.2 ± 5.5
Duration of complaint, months (mean ± SD)	52.0 ± 93.2	33.3 ± 52.3	49.9 ± 84.2	56.3 ± 129.1	95.8 ± 143.2
Baseline pain (NRS 0-10, mean ± SD)	5.1 ± 2.5	5.1 ± 2.4	5.7 ± 2.5	3.0 ± 1.6	5.1 ± 2.5
Baseline ODI (0-50, mean ± SD)	24.8 ± 7.6	24.6 ± 5.3	28.7 ± 9.6	18.6 ± 2.9	23.8 ± 9.2
Sex — Male, n (%)	52 (52.5%)	25 (58.1%)	10 (35.7%)	5 (55.6%)	12 (63.2%)

### 3.3 Treatment pathways

At the 6-week follow-up, 43 patients received both pharmacologic treatment and a therapy treatment, 28 received pharmacologic therapy alone, 9 participated in a therapeutic intervention alone, and 19 received neither modality. Accordingly, four mutually exclusive treatment cohorts were defined.

### 3.4 Clinical outcomes at 6 weeks

#### 3.4.1 Patients receiving therapy and medication (n = 43)

Among patients who received both a medication and therapy at 6 weeks, the baseline duration of complaint averaged 33.3 ± 52.3 months, the baseline pain score averaged 5.09 ± 2.4, and the baseline ODI score averaged 24.6 ± 5.3. At 6 weeks, the pain score averaged 3.91 ± 2.4, perceived pain reduction 42% ± 35%, the ODI 21.8 ± 6.6, and the global perceived improvement 1.5 ± 1.8. The mean change from baseline to 6 weeks was −1.2 ± 3.00 points for pain and −2.4 ± 7.0 points for ODI. The number of therapy sessions averaged 7 ± 5.6, and there was no evidence of a statistically significant association between the number of sessions and change in NRS (Spearman r = −0.06; p = 0.71), ODI (r = −0.21; p = 0.36), % relief (r = 0.17; p = 0.33), and GPI (r = 0.16; p = 0.35).

#### 3.4.2 Patients receiving Medication Only (n = 28)

Among patients who received a medication but not therapy at 6 weeks, the baseline duration of complaint averaged 49.9 ± 84.2 months, the baseline pain score averaged 5.71 ± 2.5, and the baseline ODI score averaged 28.7 ± 9.6. At 6 weeks, the pain score averaged 3.93 ± 2.7, perceived pain reduction 32% ± 34%, the ODI 25.6 ± 8.2, and the global perceived improvement 1.0 ± 2.2. The mean change from baseline to 6 weeks was −1.8 ± 3.22 points for pain and −2.7 ± 4.8 points for ODI.

#### 3.4.3 Patients receiving Therapy Only (n = 9)

Among patients who received therapy but not a medication at 6 weeks, the baseline duration of complaint averaged 56.3 ± 129.1 months, the baseline pain score averaged 3.00 ± 1.6, and the baseline ODI score averaged 18.6 ± 2.9. At 6 weeks, the pain score averaged 3.12 ± 2.0, perceived pain reduction 54% ± 30%, the ODI 16.5 ± 4.0, and the global perceived improvement 1.8 ± 2.0. The mean change from baseline to 6 weeks was 0.4 ± 1.51 points for pain and −1.0 ± 3.0 points for ODI. The number of therapy sessions averaged 6.7 ± 4.4, and there was no evidence of a statistically significant association between the number of sessions and change in NRS (Spearman r = −0.38; p = 0.40), ODI (r = −0.93; p = 0.01), % relief (r = 0.50; p = 0.25), and GPI (r = 0.15; p = 0.75).

#### 3.4.4 Patients receiving neither treatment (n = 19)

Among patients who did not receive a medication or therapy at 6 weeks, the baseline duration of complaint averaged 95.8 ± 143.2 months, the baseline pain score averaged 5.11 ± 2.5, and the baseline ODI score averaged 23.8 ± 9.2. At 6 weeks, the pain score averaged 3.57 ± 3.4, perceived pain reduction 43% ± 48%, the ODI 17.5 ± 9.2, and the global perceived improvement 0.9 ± 2.3. The mean change from baseline to 6 weeks was −1.7 ± 3.27 points for pain and 1.0 ± 1.4 points for ODI.

#### 3.4.5 Patients who tried PT for the first time (n = 33)

For patients who were trying PT for the first time, the mean change in pain from baseline to 6 weeks was −1.0 ± 2.76 points, and the mean change in disability was −2.6 ± 5.69 points. The average perceived pain reduction at 6 weeks was 49% ± 34%, and the average global perceived improvement was 1.6 ± 2.1.

#### 3.4.6 Patients who were on opiates (n = 12)

All patients who enrolled in the study already on opiates reported being on opiates at the study conclusion. The mean change in pain from baseline to 6 weeks was −1.6 ± 4.29 points; the mean change in disability was −3.3 ± 10.39 points; the mean perceived pain reduction was 36% ± 34% (n = 12); and the average global perceived improvement was 0.6 ± 2.6 (n = 12).

#### 3.4.7 Patients who were prescribed PT and did attend (n = 37) vs those who were prescribed PT and did not attend (n = 36)

Patients who attended PT began with lower baseline pain and disability on average (NRS 4.5 vs 5.6; ODI 22.5 vs 27.9). At 6 weeks, the attending PT group reported lower NRS (3.8 vs 4.4), lower ODI (20.5 vs 25.7), higher % relief (44% vs 30%), and higher global perceived improvement (1.5 vs 0.7) (See Table 2).

**Table 2:** Comparative relief.

	NRS (6wk)	% Relief (6 wk)	ODI (6 wk)	GPIC (6 wk)	Δ NRS (6 wk vs baseline)	Δ ODI (6 wk vs baseline)
Therapy + Meds	3.91	42%	21.78	1.47	−1.2	−2.4
Meds only	3.93	32%	25.6	0.96	−1.8	−2.7
Therapy only	3.12	54%	16.5	1.75	0.4	−1.0
No treatment	3.57	43%	17.5	0.86	−1.7	1.0

## 4 Discussion

The typical participant in this prospective cohort was a 58-year-old overweight white patient with 52 months of symptoms, a baseline NRS pain score of 5/10, and an ODI reflective of severe disability. Based on treatment recommendations and patient adherence, four treatment groups were created.

At 6 weeks, pain scores across all groups converged in the low-to-moderate range, and average disability changes were small. Regimens that included medication were associated with the most consistent short-term analgesic improvement, while therapy alone showed little mean change, possibly reflecting lower baseline severity and less room to improve rather than lack of value. Patients who were prescribed physical therapy and attended finished with better absolute pain, disability, and patient-reported improvement than those who did not attend, but these differences are confounded by baseline imbalances. Moreover, the better outcomes for PT attendees additionally may be due to their milder clinical presentation rather than the PT itself. There was no evidence of a dose response with greater therapy session counts in the combined medication + therapy cohort. Practically, these data support setting expectations for modest analgesic benefit when medication is used and emphasizing attendance rather than visit frequency in the first six weeks when physical therapy is prescribed.

Several inferences are justified by these data, with appropriate caution. First, a short (6-week) course that includes medication is associated with modest average reductions in pain (about 1–2 points) and small improvements in disability, acknowledging substantial interpatient variability given the large standard deviations. Second, adding therapy to medication did not demonstrate a clear additive benefit over 6 weeks on average; this should not be taken to imply that therapy lacks value. Third, pain change does not necessarily translate into functional change, as evidenced by the no-treatment cohort. Fourth, among patients for whom physical therapy is prescribed, attendance is associated with better outcomes at 6 weeks, although causal attribution cannot be made due to baseline imbalances and nonrandom treatment pathways.

The no treatment group and the physical therapy and medication group demonstrated comparable NRS and ODI scores at both baseline and the six-week follow-up. The absence of superior outcomes in the treatment group may reflect the natural history of the condition or regression to the mean, both of which frequently account for symptom resolution independent of clinical intervention. However, the lack of randomization and the disparity in cohort sizes preclude definitive comparative conclusions regarding treatment efficacy. Selection bias may further confound these results, as the no treatment group may have possessed distinct psychosocial profiles or utilized unmeasured self-management strategies. Consequently, while these data suggest that observational recovery may parallel formal intervention in certain contexts, randomized controlled trials are required to isolate the specific therapeutic effects of therapy and medication. These findings underscore the necessity of accounting for alternative factors in the management of low back pain.

### 4.1 Limitations

This study has several limitations. First, the observational design resulted in non-randomized treatment pathways, leading to baseline imbalances; specifically, patients who attended PT had lower baseline pain scores than those who did not, which confounds the association between attendance and superior 6-week outcomes. Second, the sample size (n = 99) resulted in small subgroups, particularly the "Therapy Only" cohort (n = 9), limiting the statistical power to detect differences between modalities. Finally, the follow-up was limited to the six-week mandate period, prohibiting conclusions regarding long-term efficacy.

Accordingly, between-group differences should be interpreted as real-world outcomes rather than estimates of comparative effectiveness. For practice, the data support setting expectations that short-term medication-inclusive regimens often yield modest early analgesic benefit and encouraging attendance when physical therapy is prescribed.

## 5 Conclusion

In this prospective cohort of 99 adults with low back pain, 6-week outcomes clustered in the low-to-moderate pain range with small average improvements in disability. Treatment pathways that included medication were associated with short-term analgesic gains, whereas therapy alone showed little mean change. Among patients for whom physical therapy was prescribed, those who attended had better absolute pain, disability, and patient-reported outcomes at 6 weeks than non-attenders, although these differences are confounded by baseline imbalances. There was no evidence of a dose–response with greater number of therapy sessions. The study is limited by nonrandomized treatment pathways, unequal and small subgroup sizes (especially therapy-only). The primary takeaway is that, with a 6-week conservative care treatment plan, medication-inclusive regimens and PT adherence, not higher session counts, are the most defensible early treatments.

## Funding

None.

## Conflict of interest

None.
